# Recent Advances in Carbon-Based Adsorbents for Adsorptive Separation of Light Hydrocarbons

**DOI:** 10.34133/2022/9780864

**Published:** 2022-06-21

**Authors:** Yong-Sheng Wang, Xue-Jie Zhang, Ya-Qi Ba, Tian-Yi Li, Guang-Ping Hao, An-Hui Lu

**Affiliations:** State Key Laboratory of Fine Chemicals, Liaoning Key Laboratory for Catalytic Conversion of Carbon Resources, School of Chemical Engineering, Dalian University of Technology, Dalian 116024, China

## Abstract

Light hydrocarbons (LHs) separation is an important process in petrochemical industry. The current separation technology predominantly relies on cryogenic distillation, which results in considerable energy consumption. Adsorptive separation using porous solids has received widespread attention due to its lower energy footprint and higher efficiency. Thus, tremendous efforts have been devoted to the design and synthesis of high-performance porous solids. Among them, porous carbons display exceptional stability, tunable pore structure, and surface chemistry and thus represent a class of novel adsorbents upon achieving the matched pore structures for LHs separations. In this review, the modulation strategies toward advanced carbon-based adsorbents for LHs separation are firstly reviewed. Then, the relationships between separation performances and key structural parameters of carbon adsorbents are discussed by exemplifying specific separation cases. The research findings on the control of the pore structures as well as the quantification of the adsorption sites are highlighted. Finally, the challenges of carbonaceous adsorbents facing for LHs separation are given, which would motivate us to rationally design more efficient absorbents and separation processes in future.

## 1. Introduction

Light hydrocarbons (LHs, e.g., C_1_-C_4_ hydrocarbons) are basic feedstocks for fuels, plastics, and polymers [1–3]. The separation of LHs with similar physical and chemical properties is one of the most important but challenging petrochemical processes [4]. The dominated thermal-driven separation technology by the difference in boiling points (i.e., distillation) suffers from high energy consumption, which accounts for up to half of the total energy consumption in the chemical industry [1]. The adsorptive separation based on advanced adsorbents is a promising alternative for LHs separation because of the advantages in terms of low energy consumption, environmental friendliness, and easy regeneration [5–8].

Table 1 displays the physicochemical parameters of LHs of C_1_ to C_4_ and the background for their separation. Methane (CH_4_) is produced either from petrochemical sites or unconventional gas sources (e.g., coalbed methane and shale gas). The gas mixture contains a couple of impurities, mainly including N_2_, CO_2_, and a small fraction of C_2+_ hydrocarbons in some cases. The highly valuable C_2-4_ olefins are mainly produced by the steam cracking of feedstocks from gaseous resources, such as ethane (C_2_H_6_) and propane (C_3_H_8_), to liquid, heavier feedstocks, such as naphtha, gas oil, etc. The outlet stream is a gas mixture composed of mainly paraffin and olefin. Accordingly, the relevant separation includes CH_4_/N_2_, CH_4_/CO_2_, and CH_4_/C_x_H_y_ separation for natural gas purification; C_x_H_2x_/C_x_H_2x+2_ for the production of high-purity C_2-3_ olefins; and saturated/unsaturated C_4_ isomer separation for producing the precursors for synthetic rubbers and additives for high-octane gasoline.

In the typical adsorptive separation process, the gas mixture flows into the adsorption column, and the weakly adsorbed adsorbate is preferentially drained out from the adsorption column. The adsorption separation process can be divided into separation and purification [9–11]. For the former, the separated gas components (i.e., the strongly adsorbed adsorbate) account for a large proportion of the mixture [12, 13]. For the latter, impurities (usually less than 2%) are more strongly adsorbed to obtain high-purity gas. For instance, the removal of acetylene (C_2_H_2_, about 1%) from the mixture of C_2_H_4_/C_2_H_2_ to upgrade the C_2_H_4_ stream is the so-called purification process [14, 15]. No matter which type of adsorption separation process, separation efficiency can be affected by the interactions between adsorbate and adsorbent and between different adsorbates, while the optimization of interactions mainly relies on the structural modification of the adsorbent.

**Figure 1:**
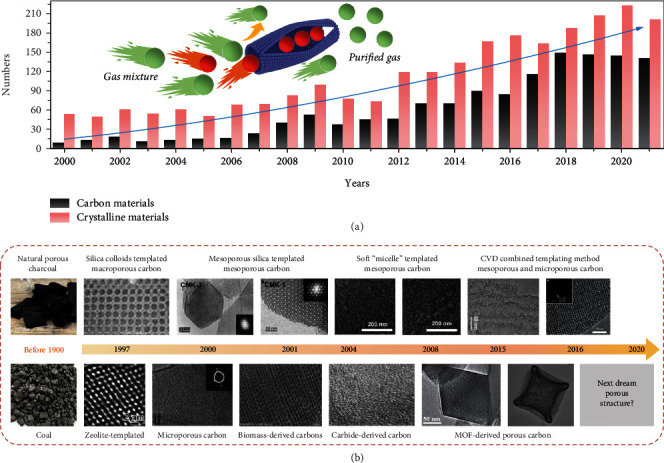
(a) The development of the field of “carbon materials” and “crystalline materials” on light hydrocarbons separation in the last two decades (web of science until March. 2022). (b) The research timeline on the development of porous carbons with defined pore structures.

With the advances in new porous solids, the adsorptive LHs separation is becoming more effective and playing a bigger role in molecular separation.  Figure 1(a) shows the development of advanced adsorbents for LHs separation in the past two decades, which is exemplified by carbon materials and crystalline materials (e.g., MOFs and zeolites) [16]. Crystalline adsorbents have the advantages of well-defined pore structures and highly uniform micropore size. A typical example is zeolite material with 3D cage cavities. The charged skeletons or open metal sites provide tunable binding affinities towards unsaturated hydrocarbons and thus enable well control over a variety of host–guest interactions [4, 16]. However, the higher prices of raw materials and the stability of the crystalline adsorbents limit the industrial application of these materials. Only adsorbents with a good match between performance and cost are preferred for industrial production.

Porous carbons hold promises as adsorbents for LHs separation because of their inherent advantages such as structural robustness, low cost, and high porosity [17–20]. Unlike crystalline materials, porous carbons hold promises as adsorbents for LHs separation because of their inherent advantages such as structural robustness, low cost, and high porosity. Structurally, porous carbons exhibit a unique slit-shape microporous structure, which is built from disorganized graphene-like planes, forming a tightly stacked structure with chemically stable and acid/base resistance [21, 22]. These disorganized, multimodal micropores usually limit the tunability of the pore structure at the sub-Ångstrom scale. In addition, the surface of most carbons is predominantly nonpolar, reflecting the highly hydrophobic property due to high-temperature treatment. After decades of development, researchers have been able to relatively well control the pore size and geometries (Figure 1(b)) for targeted separations. The common strategies include the judicious selection of polymer precursors, control of the carbonization temperature, and pre- or postfunctionalization [23–25]. Not only the tunable pore structure but also the rich controllability of surface chemistry also brings more possibilities to porous carbons [26, 27]. The diverse chemical sites of porous carbon show different binding affinities to adsorbate molecules and followingly different guest-host interactions [28, 29]. For instance, the oxygen-containing groups on porous carbons can improve the surface polarity and enhance the affinity for C_2_H_6_ [30]. Alternatively, the Ag(I) and Cu(I) species can interact strongly with olefins by *π*-*π* interactions [31, 32]. Apart from the above two points, porous carbons show high processability so that they can be engineered to a specific morphology or readily combine with other functional materials for advanced composite adsorbents [33]. By applying these strategies, porous carbons have proven to be powerful adsorbents for the separation of LHs.

Continuous progress has been made over porous carbon-based adsorbents in improving adsorption capacity, selectivity, mechanical stability, and so on. In order to promote the rapid development of carbonaceous adsorbents in the field of light hydrocarbon separation, it is necessary to summarize and sort out the structure-effect relationship of porous carbon. This motivates us to organize this review to bring potential readers a full picture of the adsorptive separation of LHs from synthesis protocols of porous carbons to their structural properties as well as performance assessments. In this review, we overviewed the separation mechanism and the preparation protocols and discussed the latest advances in LHs separation. Exemplified with typical LHs separation cases, we highlighted the correlations between the structural parameters of porous carbons and their separation performances. Furthermore, we emphasized the targeted design of carbonaceous adsorbents on the molecular level for ideal LHs separation performances. At last, we discussed the challenges and the research opportunities for the adsorptive separation of hydrocarbons in future.

## 2. Separation Mechanism

The separation of LHs is mainly achieved by distinguishing the diffusion rate and/or the adsorption affinities. Typically, the adsorption separation of LHs mechanism can be divided into three cases: thermodynamic effect, kinetic effect, and molecular sieving effect. Each mechanism was discussed as follows.

### 2.1. Thermodynamic Effect

**Figure 2:**
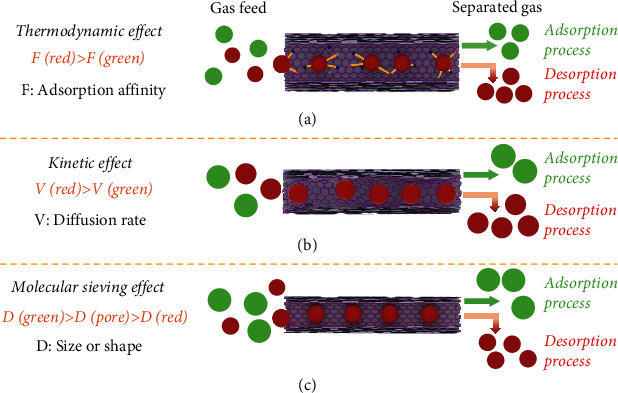
Proposed mechanisms for the adsorptive separation of LHs. (a) Thermodynamic effect. (b) Kinetic effect. (c) Molecular sieving effect.

Thermodynamically, the separation performance is based on different interaction forces between adsorbent and adsorbate [34, 35] (Figure 2(a)). The adsorbents usually contain gas-philic sites such as open metal active sites to selectively adsorb olefin by *π*-*π* interactions [36, 37]. Notably, the olefins have higher polarity than paraffin, which also leads to preferential enrichment of olefins molecules on the polar sites of porous carbon. More specifically, the surface functional groups or defective edge structures in porous carbons would show electric dipole or quadrupole interactions with guest LHs molecules [16].

### 2.2. Kinetic Effect

The gas separation by porous carbons is mostly dominated by the kinetic effect. The kinetic separation depends on different gas molecular diffusion rates of adsorbates traveling into the pores [38, 39] (Figure 2(b)). In most cases, the diffusion rates of olefins are faster than paraffins, because olefins have a lower molecular weight and boiling point than paraffins [40, 41]. Compared with the thermodynamic effect, the kinetic effect allows a low energy consumption for product recovery, because of the absence of affinity between LHs and adsorbents. As a result, the kinetic effect is more favorable for industrial processes. For clarity, the single-component adsorption isotherm is not suitable for determining a material whether achieves the LHs separation by kinetic effect or not. In general, time-resolved gas adsorption is typically used to verify the kinetic effect by comparing the diffusion constants of different gases. The diffusion time constants, which are obtained by fitting kinetic adsorption profiles, are the standard to evaluate the rate of diffusion.

### 2.3. Molecular Sieving Effect

The molecular sieving is an outcome of size/shape exclusion, which is the ideal case for gas separation [42] (Figure 2(c)). It should be mentioned that the molecular sieving effect can be considered a kinetic effect at the boundary conditions. In general, olefins have a smaller size than paraffin, when they have the same number of carbon atoms. Therefore, olefin can quickly enter the micropores, while paraffin is completely shielded from adsorption. However, if the micropore size is similar to the size of the adsorbate, it will cause a slow diffusion of the adsorbed component. Since the overall configuration of micropores is built from disorganized graphene-like planes in the porous carbons, the formation of uniform micropores is a highly challenging task. Although there are available quite a few kinds of carbon molecular sieves for the separation of CO_2_/N_2_ and H_2_/CO by the molecule sieving effect, it remains scarce for the time being to achieve the LHs separation based on the molecule sieving effect.

The gas adsorption separation is a complicated process, resulting in multiple separation mechanisms synergetic effect in most cases [43]. For instance, the micropore walls with size sieving properties are loaded with open active metal or doped heteroatoms. On the one hand, the size of the micropores can achieve LHs separation based on kinetics effect or molecular sieving effect; on the other hand, the adsorption sites on the pore walls can strengthen the target molecule interactions. Notably, practical separation processes are mainly controlled by synergistic thermodynamics and kinetic effects. The calculation of the combined selectivity is based on gas diffusivities and Henry's constants [44].

## 3. Strategies on Structural Modulations

Based on the three separation mechanisms above, the synthesis and modification of porous carbons can be controlled in a targeted manner. For thermodynamic effect, the adsorbent is required to create adsorption sites by doping heteroatoms and loading open metal active sites. In the case of molecular sieving effect and kinetic effect, it depends on the fitting pore size and pore geometry of the adsorbent. In addition, the adsorption capacity is closely related to the surface chemistry and the pore structure of the adsorbent. Thus, this section summarized the synthesis and modification strategies for high-performance carbonaceous adsorbents. Meanwhile, the relationship between pore structure, surface chemistry, and adsorbent capacity is discussed.

### 3.1. Tuning of Chemical Properties

The regulation of surface chemistry can achieve LHs separation by thermodynamic effect. In general, most porous carbons are prepared by carbonizing precursors in an inert atmosphere. The higher the carbonization temperature, the fewer polar functional groups remain on the carbon surface. Thus, carbon materials are inherently non-polar, and not suitable for LHs separation by thermodynamic effect. It is highly challenging to render a non-polar surface to be highly polar. Typically, the main methods of regulating surface chemistry for porous carbons include doping heteroatoms and loading open metal active sites such as Ag(I) and Cu(I). We will discuss the above methods of porous carbons preparation in more detail in the following.

#### 3.1.1. Heteroatom Doping

Heteroatom doping is to replace some carbon atoms in the basal plane or edges of graphene layers with noncarbon atoms. It obviously changes the electron and charge properties of porous carbons; thus, it can tune their polarity for enhanced interaction between the carbon pore walls and the C=C/C≡C [45]. In principle, heteroatom doping can be carried out in two ways. One is the post-treatment, in which the pristine carbons are functionalized by surface reactions using dopant gases under optimized conditions. Dopant gases include NH_3_ for N-doping, H_2_S for S doping, and Cl_2_ for Cl doping [46–50]. Normally, the gas phase functionalization occurs at high temperatures (mostly 600-900 °C). Such harsh treatment sometimes leads to further shrinkage of the porous structure and reduction of the accessible pores. In some cases, the doping was found to be limited in the surface region, unlikely effective for bulk phase functionalization [51].

The other one is in-situ doping. In this strategy, the precursors, both amorphous and crystalline, are heteroatom-rich [52]. The amorphous precursors include phenolic resin, polyaniline, and polybenzoxazine [18]. The crystalline precursors show long-range order, which, in some cases, will be partly remained during carbonization. These would result in pores with a high-level heteroatom doping in both ways [53]. Besides, using various biomaterials as precursors is a common method for heteroatom doping [20]. It should be noted that the carbonization temperature will affect the degree of doping [18, 54]. Since the heteroatom is inherited from the precursor, the generated functional groups are more stable than the doping posttreatment. Overall, the in situ doping strategy is quite feasible and holds great potential for scaling up. Furthermore, doping with dual even multi-heteroatoms can be imparted, which would be fascinating for task-specific LHs separation.

#### 3.1.2. Open Metal Active Sites

The decoration of Ag(I) or Cu(I) on the pore wall of adsorbents favors the trapping of olefin over paraffin due to *π*-*π* interactions between open metal active sites and *π*-bond of olefin. Generally, the metal species are adsorbed, reduced, and anchored to the sites of vacancies or defects of the porous carbons [55, 56]. In addition, the sulfurized porous carbons can improve the loading efficiency of Ag(I), because of the high affinity of the Ag(I) to the sulfur functionalities [31].

### 3.2. Tuning of the Porous Structure

According to IUPAC standard, the pores can be divided into macropores (>50 nm), mesopores (2-50 nm), and micropores (<2 nm) [57]. Among them, the micropore size is the dominant factor affecting the molecular sieving effect and kinetic effect. For most porous carbons, the overall configuration of micropores is built from disorganized graphene-like planes, normally composed of narrow slit-shaped apertures (<0.7 nm) and layer stacked nanovoids in the length scale of 0.7-2 nm [58]. Excellent selectivity will result from the size of the narrow micropore size distribution which falls right between the kinetic diameters of gas mixtures, enabling a sieving effect. A narrow micropore size distribution can also enhance the adsorption capacity in low pressure regions. However, the gas diffusion in the highly concentrated ultramicroporous channels is suppressed, leading to sluggish adsorption kinetics. The mesoporous structures are evidenced as an effective pathway for gas molecules diffusion [59]. Therefore, by adjusting pore sizes (e.g., the combination of mesopores and micropores), it is possible to achieve the fast transport of LHs and effective LHs separation performance. According to the literature, a close relationship between the formation of micropore structure and polymer precursors, pyrolysis conditions, and treatment methods has been figured out [60, 61]. We will discuss the pore structure control method of the porous carbons in more detail in the following.

#### 3.2.1. Precursor Selection

The judicious choice of precursors is crucial for obtaining porous carbons with well-defined ultramicropores. Porous carbon derived from biomass, such as coal, wood, and fruit shells, can change pore size by changing carbonization conditions, but this strategy still does not precisely control ultramicropore size [62]. On the contrary, solution chemistry based on molecular level design is more effective, and a series of precise micropore regulation rules of porous carbon has been developed. It is different from the traditional process for the preparation of porous carbons by the solid-state method. Precisely distributed ultramicropores are created essentially by thermal transformation of cross-linked polymer networks, followed by the release of volatile fragments, dynamic aromatization, and alignment of the conjugated *sp^2^*-C-based microcrystalline domains [63]. Thus, the pore size distribution is also highly relevant to the carbon chain structure of the polymer precursors [64]. As a consequence, it is important to choose suitable crosslinked polymeric structures, which include but are not limited to polyacrylonitrile, polyvinylidene dichloroethylene, polyimide, phenolic resin, poly(ionic liquid)s, polythiophene, and polydopamine. For instance, Lu and co-workers reported that a novel type of ultramicroporous carbon sheets was obtained by using phenolic resin-based precursors with a hundred-nanometer-thickness, which showed narrow bimodal micropore distribution centered at 0.48 and 0.82 nm, respectively [64]. In addition, the influence of the aliphatic chain length of the primary amine moieties on the micropore structure of 2D polybenzoxazine-based porous carbons was also investigated by Lu and co-workers [65]. The result showed that the shorter the length of aliphatic amine moieties, the higher ordering of the stacking of graphene layers, and the higher the unimodality in micropore size achieved. Besides, the MOFs-related materials, which possess tunable pore size and surface chemistry, are sacrificial templates to produce micropores carbons [66, 67].

#### 3.2.2. Treatment Method

**Figure 3:**
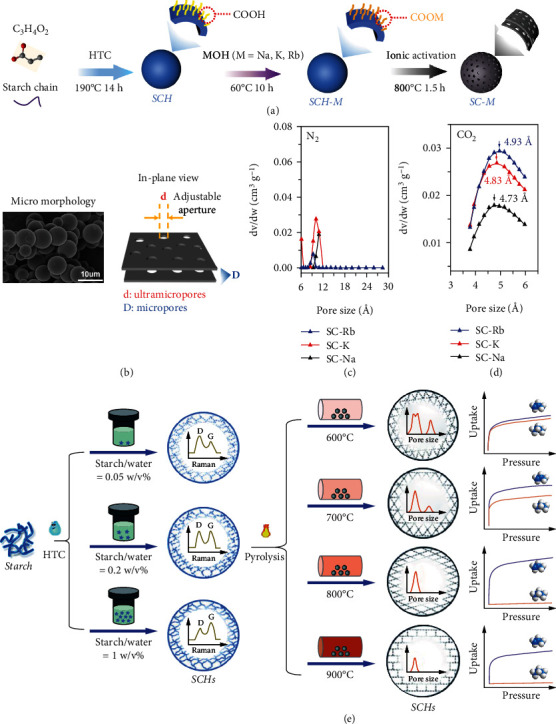
(a) The ultramicropore formation process for the starch-based carbon adsorbents (SC-M) by using the alkali metal ion-exchange method. (b) The schematic diagram of the microstructure. (c, d) Pore size distribution of SC-M [70]. (e) Synthesis schematic of starch-based carbon molecular sieve (SCMS) [71].

Besides the selection of precursors, the pre-/post-treatment methods can effectively control the pore size of porous carbon. To date, a series of reliable treatments have been successfully developed, such as chemical activation, chemical vapor deposition (CVD), and water vapor activation [68]. As a typical pretreatment method, the chemical activation method can obtain ultramicroporous carbon by using alkali hydroxide or salts containing alkali ions [69]. For instance, Xiao and co-workers prepared a series of ultramicroporous starch-based carbon spheres (SC-M; M = Na, K, Rb), which could achieve sub-Ångstrom tunable ultramicropore apertures by using the alkali metal ion-exchange method. Remarkably, the ultramicropore sizes of SC-Na, SC-K, and SC-Rb are concentrated at 4.73, 4.83, and 4.93 Å, respectively. This indicates that the ultramicropore size of carbon can be finely tuned by activation using different sizes of metal ions. Among the materials, SC-K exhibits excellent C_3_H_6_/C_3_H_8_ separation performance by molecular sieving effect, and adsorption capacity ratios for C_3_H_6_/C_3_H_8_ is 5.36 [70] (Figures 3(a)–3(d)).

The experimental parameters, such as dosage, the ratio of raw materials, and activation temperature, are critical for micropore production in the pre-treatment. For instance, Xiao and co-workers controlled the concentration of starch hydrothermally to tune the size of micropores (SCH-X-Y, X = the starch/water ratio of 0.05, 0.2, and 1 w/v%, Y = different pyrolysis temperatures) [71]. Different starch hydrothermal concentrations can affect the graphitization and defect ratio of the carbon material, which was verified by Raman (Figure 3(e)). The I_D_/I_G_ values of SCHs followed the order SCH-0.05 > SCH-0.2 > SCH-1. Among them, the low I_D_/I_G_ value of the SCH-1 reflected relatively low lattice edges or defects on plane terminations of disordered graphite. The author analyzes that the higher starch concentration can produce more hydroxymethyl furfural and organic acids that would generate fewer defects of hydrochar in the polymerization and aromatization process, resulting in improvement in aromatic microdomains and microcrystallite size of SCHs. It is crucial for synthesizing carbon molecular sieves with uniform pore size. In addition, higher pyrolysis temperature rearranges the carbons toward graphitization and orderliness. Thus, the (002) crystal planes sharpen gradually with increasing pyrolysis temperature. Meanwhile, the micropore size reduces to about 4.78 Å which is between the kinetic diameter of C_3_H_6_ and C_3_H_8_. The CVD as a typical posttreatment method is an extraordinarily effective strategy to reduce the pore size of the porous carbons through the deposition of pyrolytic carbon from the hydrocarbon. However, it will reduce accessible micropore volume and inevitably decrease the adsorption capacity [72, 73]. Alternatively, the exploration of novel treatment methods and a flexible combination of different treatment methods will have effective ways to control the microporous size of porous carbons for efficient LHs separation in future.

### 3.3. Adsorption Capacity

The adsorption capacity is related to the surface chemistry and the pore structure of an adsorbent. Thermodynamically, the adsorbent has active sites that ensure moderate interactions with adsorbates, while it does not mean the more active sites, the higher the adsorption capacity. For instance, Deng et al. found that N content showed limited influence on the regression model by studying the regression models of pore volume under specific pore size and CO_2_ capacity [74]. At low pressure of 0.15 bar, the N-doping played a dominant role. A content of 5.1 wt% led to two folds higher CO_2_ capacity than that with N content of 1.5 wt%. However, at 1.0 bar, the CO_2_ capacity is dependent on the micropore volumes. Schwartz et al. conducted theoretical studies exemplifying CH_4_ as a gas probe [75]. In their case, the mean CH_4_ adsorption energies were found lowered due to the higher degree of oxidation over carbon adsorbents, which reduced the overall CH_4_ uptake as compared to the unoxidized surface.

The functional groups were also found beneficial to increase the adsorption capacity at high temperatures [62]. However, the strong binding sites will inevitably generate a large amount of adsorption heat during the adsorption process, resulting in a decrease in the adsorption capacity. In addition, for strong adsorption interactions, much lower pressures or higher temperatures are required for adsorbent regeneration in PSA and TSA processes. As a result, a balanced regulation of pore structure and surface chemistry can be used to achieve optimized performance for a specific separation process.

## 4. Separation Based on Thermodynamic Effect

As discussed above, huge attention has been paid to the development of novel porous carbons for LHs separation. However, as the opposite process of mixing, separation is generally not a spontaneous procedure, because it is a violation of the second law of thermodynamics. Consequently, gas separation often requires adsorbents with a considerable number of adsorption sites, strong guest-host interactions, and a large accessible surface area. In this section, we overviewed the porous carbon adsorbents applied in thermodynamic effect for LHs separation.

### 4.1. CH_4_

With a high heat of combustion (55.7 kJ/g), CH_4_ accounts for 22% of the global fuel consumption, and this value is expected to increase continuously in the future. Therefore, as a substitute for traditional fossil fuels, it is urgent to develop and utilize CH_4_ sources [76]. Natural gas is composed of CH_4_ and the predominant impurity of N_2_ and CO_2_, which must be separated. The CH_4_/N_2_ and CH_4_/CO_2_ separation performance of selected carbon-based adsorbents are summarized in Table 2. The porous carbon for CH_4_ purification is mainly divided into three types: biomass carbon, polymer-based carbon, and pitch-based carbon. Bio-based and polymer-based derived carbons can better achieve heteroatom doping, resulting in affinities for different gases. Among them, the polymer-derived carbon can achieve not only more controllable morphologies, such as carbon sheets and carbon spheres, but also more concentrated pore size distributions. For the capture of low concentration CH_4_, improving the selectivity and adsorption capacity of CH_4_ is the main task for the adsorbent. However, the adsorption capacity of CH_4_ is difficult to exceed 2 mmol g^−1^, because the CH_4_ has a low polarizability (2.6 × 10^−24^ cm^3^) and boiling point (-161 °C) as well as no quadrupole moment, so it is difficult to achieve effective adsorption of CH_4_ in ultramicropores at 25 °C. Although the high specific surface area of porous carbon can increase its adsorption capacity of CH_4_, the adsorption capacity of other gas will also increase, resulting in a decrease in selectivity. Probably, the preparation of high-density ultramicroporous channels conforming to the molecular dynamics-based model of CH_4_ is important for the construction of large-capacity and high-selectivity porous carbon adsorbents.

#### 4.1.1. CH_4_/CO_2_

In this section, we discussed the latest efforts from the materials aspect, i.e., the design of novel carbon adsorbents for CO_2_/CH_4_ separation. The presence of H_2_O and CO_2_ in natural gas will corrupt the transportation and storage system. To meet the specifications for fuel gas, CO_2_ must be removed. To date, many porous carbons showed excellent CO_2_ adsorption performance. However, due to the similar kinetic diameters of CH_4_ and CO_2_, it is still challenging for their efficient separation. In previous reports, both microporous structure and heteroatoms doping are beneficial for CO_2_ adsorption, specifically ultramicropores (<1 nm) and N doping. The ultramicropores not only provide storage space for CO_2_ but also confine CO_2_ by Van der Waals' forces. The N doping can increase the surface polarity and basicity, which achieves weak chemical interaction between CO_2_ and the pore wall. However, does the CO_2_ capture capacity increase with the increase of basic sites? To verify this issue, Deng and co-workers prepared three samples with almost identical pore structures and varied N content (6.78%, 4.81%, and 0.38%) from the oil-tea seed shell (OTSS) through direct activation with solid NaNH_2_, which acts as both porogen and nitrogen source [83]. The CO_2_ adsorption isotherms showed a nearly indistinguishable shape and adsorption performance at both 1 and 0.15 bar. In this case, the N-doped functional groups merely affected the CO_2_ adsorption behaviors. Besides, OTSS-2-550 with the largest micropore volume (0.63 cm^3^g^−1^) displayed the highest CO_2_ uptake of 5.65 mmol g^−1^ at 0 °C and 100 kPa. This revealed that the microporous structure is rather critical in contributing to the CO_2_ adsorption capacity. In addition, to further explore the effects between micropores and N-doped functionalities on CO_2_ adsorption. Deng and co-workers developed N-doped (ca. up to 5.11 wt%) microporous carbons (SNMC-X-Y, *X* = the mass ratio of KOH/SFRH =1/1, 2/1, and 3/1; *Y* = the activation temperature) by using an N-rich polymer as a precursor. The precursor was made from the polymerization of resorcinol and hexamethylenetetramine by using a one-pot melting-assisted and solvent-free method [74]. By studying the regression models of pore volume under specific pore size and CO_2_ capacity, the authors found that both high and low N content showed limited influence on the regression model. But the SNMC-1-600 with N content of 5.11 wt% showed about two folders higher CO_2_ capacity than that of SNMC-2-700 (1.5 wt%) at 0.15 bar. It implies that the CO_2_ capacity at 1 bar is dominated by micropores volumes rather than the amount of N-doped functional groups, while N content remarkably influences the CO_2_ capacity at 0.15 bar. Additionally, SNMC-3-800 with the highest S_BET_ (3657.0 m^2^g^−1^) showed excellent CO_2_ capacity up to 22.06 mmol g^−1^ at 20 bar and 25 °C, due to the highest KOH/SFRH ratio and pyrolysis temperature. However, the authors only studied the storage of CO_2_ and did not study CO_2_/CH_4_ separation performance under high pressure.

For the pressure swing adsorption (PSA) process, it is essential to explore the gas separation performance under high pressure. Li and co-workers prepared the N-doped porous carbon spheres (Glc-C-4) with a high S_BET_ of 3153 m^2^ g^−1^ via hydrothermal carbonization and then KOH activation [90]. The Glc-C-4 had a narrow pore size distribution and rich N-doped active sites, which could be used as an adsorbent for capturing CO_2_. The Glc-C-4 exhibited a high CO_2_ capacity (22.4 mmol g^−1^) and relatively low CH_4_ capacity (11 mmol g^−1^) under 298 K and 30 bar. With the increase of pressure, the heat of adsorption of CO_2_ increased gradually, while the heat of adsorption of both CH_4_ and N_2_ decreased, because of the energetical heterogeneity of adsorption sites for CH_4_ and N_2_. Thus, the separation selectivity was optimized, reaching 4.5 for CO_2_/CH_4_ (50 : 50 v:v) binary mixtures under 298 K and 30 bar.

#### 4.1.2. CH_4_/N_2_

In this section, we will discuss several designed carbonaceous adsorbents for CH_4_/N_2_ separation. Although the chemical and physical activation methods are quite mature to enlarge the surface area and impart additional micropores of porous carbon, it still lacks effective control over the pore size at the microporous scale for the CH_4_/N_2_ separation performance. To solve this problem, it is essential to understand the separation mechanism of CH_4_ and N_2_ in porous carbons. Kumar and co-workers studied the effect of porous structure and pore geometry of carbonaceous adsorbents on the separation of dilute CH_4_ from CH_4_/N_2_ mixture [91]. Four kinds of nanostructured carbons were selected as model adsorbents, which include the theoretical carbon structures with ideally slit or tubular pores, the realistic porous carbons with disordered pore structures, and carbon foam structures. The Grand Canonical Monte Carlo (GCMC) simulation results showed that the pore structure could affect the CH_4_/N_2_ selectivity or the adsorption behavior. More specifically, they found that the nanotube-like pores with a confined pore size exhibited a CH_4_/N_2_ separation selectivity of 2.0 at the pressure range from 6 to 8 bar, while the microporous carbon with a slit-pore and carbon foam structures showed a slightly higher selectivity of ~3.0 at lower pressures. In summary, carbon adsorbents are hard to achieve high separation performances based on the thermodynamic effect.

In recent research, it is reported that some ultramicroporous carbons displayed excellent CH_4_/N_2_ separation performance [92]. However, the gas diffusion in the highly twists ultramicropores is restrained; thus, the adsorption capacity and adsorption kinetics need to be reinforced. To overcome the diffusion limitations, the conventional strategy is to build a hierarchical pore system by imparting meso/macropores. However, Lu and co-workers show a distinct and effective method by pillaring up the 2D ultramicroporous carbons nanoplates (PCNPs) through highly processible synthesis [77]. The pillared polymer nanoplates are based on a multicomponent sequential assembly, in which the protrusions were in situ grown on the soft 2D templates (self-assembly of triblock copolymers and stearic acid) by inducing sequential condensation of phloroglucinol, terephthalaldehyde, and p-phenylenediamine (p-PDA). The authors have demonstrated that PCNPs showed fast kinetics and high selectivity. In addition, not only the areal density but also the height of the protrusions can be regulated continuously by varying the concentration of p-PDA. At a low partial pressure of CH_4_, the PCNPs showed a high CH_4_/N_2_ selectivity up to 24, and the CH_4_ diffusion constant (1.18∗10^−3^s^−1^) was two orders of magnitude faster than the commercial carbon molecular sieves (CMS, Takeda 3kt, 1.19∗10^−5^s^−1^). The PCNPs with dense ultramicropores by nanoprotrusions greatly improved diffusion rate and resulted in selective separation of CH_4_/N_2_.

#### 4.1.3. CH_4_/C_x_H_y_

**Figure 4:**
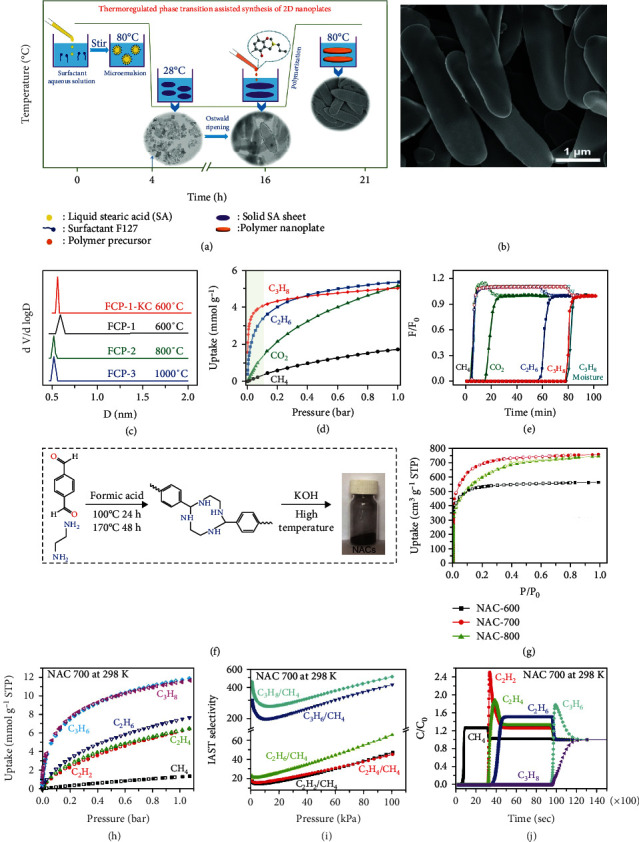
(a) Schematic illustration of the synthesis for the polymer nanoplates. (b) SEM image of FCP-1. (c) Pore size distribution. (d) Gas adsorption isotherms and (e) breakthrough profile of x/CH_4_ (10/90 v/v, X = CO_2_, C_2_H_6_, C_3_H_8_) of FCP-1-KC at 298 K and 1 bar [63]. (f) Schematic illustration of the synthesis process. (g) N_2_ adsorption isotherms. (h) LHs adsorption isotherms. (i) IAST selectivity and (j) breakthrough simulation profile in an equimolar 6-component CH_4_/C_2_H_2_/C_2_H_4_/C_2_H_6_/C_3_H_6_/C_3_H_8_ mixture [93].

In addition to CO_2_ and N_2_, the small fractions of LHs, such as C_2_H_2_, C_2_H_4_, C_3_H_6_, and C_3_H_8_, are also common impurities in natural gas. Thus, the separation of CH_4_/C_x_H_y_ has also been explored extensively. In the aspect of material design, one way is to achieve a suitable pore structure; the other is a surface modification by heteroatom doping. For instance, Lu and co-workers devised an innovative kind of 2D flat carbon nanoplates (FCP), which was synthesized by stearic acid thermoregulated phase-transition approach [63]. The liquefied stearic acid (SA) at 80 °C was dispersed into F127 aqueous solution to form a uniform microemulsion, and then the temperature was lowered to 28 °C to change the SA from liquid to solid. After Ostwald ripening, the sheet SA template is obtained (Figures 4(a) and 4(b)). The FCP showed greatly well-organized and accessible ultramicropores preferentially orientated growth of more than 80% *sp^2^-C* and confined thickness. Remarkably, the pore sizes are uniformly concentrated at 0.53, 0.56, or 0.58 nm at different carbonization temperatures of 1000, 800, and 600 °C, respectively (Figure 4(c)). The author found that the carbon crystallites were stacked together into parallel multilayers at the regions close to the sheet boundary by using Brownian dynamics simulation and TEM. Thus, the 2D structure makes the parallel oriented growth and stack of carbon crystallites, achieving the single size of the micropores. For CH_4_ purification, the FCP exhibited excellent capacity of CO_2_ (5.2 mmol g^−1^), C_2_H_6_ (5.3 mmol g^−1^), and C_3_H_8_ (5.1 mmol g^−1^) (Figure 4(d)). The order of heat of adsorption is C_3_H_8_ > C_2_H_6_ > CO_2_ > CH_4_, exhibiting the same sequence as the molecular polarizabilities. It is indicating increased host-guest interactions originating from short-range attractive forces in uniform ultramicropores. The breakthrough experiment exhibited that FCP could efficiently distinguish natural gas mixture (x/CH_4_,10/90 v/v, x = CO_2_, C_2_H_6_, and C_3_H_8_) (Figure 4(e)). In addition, their tested breakthrough performance under wet conditions: C_3_H_8_/CH_4_/H_2_O (10%/87%/3%, v/v/v). The breakthrough results reveal that the separation performance of carbon nanoplate is not affected by the presence of moisture, indicating good moisture resistance. Thus, the construction of 2D structure porous carbon is a promising method to prepare high-efficiency carbonaceous adsorbents with narrow micropore size distribution.

As for carbon matrix modification by heteroatom doping, N-doping is an effective strategy for improving gas adsorption capacity and separation selectivity. For instance, Deng and co-workers reported one kind of N-doped (ca. 3.6 wt%) activated carbons by using an N-rich polymer as a precursor, which was made from the polymerization of terephthalaldehyde and ethylenediamine [93] (Figures 4(f) and 4(g)). The NACs showed a high C_2_ and C_3_ capacity of 7.59 and 11.77 mmol g^−1^, respectively. This material exhibited a high separation selectivity of 66 for C_2_/C_1_ and 502 for C_3_/C_1_ under mild conditions (Figures 4(h) and 4(i)). As expected, the breakthrough experiments in the 6-component mixture (CH_4_/C_2_H_2_/C_2_H_4_/C_2_H_6_/C_3_H_6_/C_3_H_8_) demonstrate that the separation can be realized (Figure 4(j)). The authors believed that N functionalization played a key role in enhancing the adsorption capacity and selectivity. On the one hand, the N-doping led to stronger interactions with those easily polarized hydrocarbons; on the other hand, the pore size was optimized simultaneously.

### 4.2. C_2_H_4_/C_2_H_6_

The exploration of high-performance carbonaceous adsorbents is underway for C_2_H_4_/C_2_H_6_ separation.  Table 3 compared the selective adsorption properties of the carbonaceous adsorbents, in which the capacity, the adsorption heat, and the IAST selectivity were compared. Notably, the adsorbents without open metal sites all exhibit the property of preferentially adsorbing C_2_H_6_ in the C_2_H_4_/C_2_H_6_ separation. For this unique phenomenon, researchers have made various explanations from the perspective of pore size and chemisorption site, which will be reviewed next. However, why are there no carbonaceous adsorbents without adsorption sites on the preferential adsorption of C_2_H_4_? This topic is an opportunity for carbon scientists.

In the thermodynamic effect, the strong host-guest interactions between open metal active sites and C_2_H_4_ result in a distinct difference of porous carbon in adsorption affinity for C_2_H_6_ and C_2_H_4_. For instance, Jiang and co-workers synthesized ordered mesoporous carbon and then incorporated the CuCl species by solid-state grinding [32]. After thermal treatment, the CuCl nanoparticles were high dispersed, with no metallic Cu nanoparticles observed. The adsorbent can selectively adsorb C_2_H_4_ via *π*-*π* interactions, and the selectivity increases with the increasing loading of CuCl. Similarly, Gao and co-workers prepared the Cu(I) decorated activated carbon (AC) [95]. They first introduced the CuCl_2_ that was reduced to CuCl by thermal treatment under N_2_ atmosphere. The obtained CuCl/AC adsorbents achieved a C_2_H_4_ uptake of 2.57 mmol g^−1^ at 30 °C and 1 bar. The C_2_H_4_/C_2_H_6_ separation selectivity reached 69. Notably, it is necessary to construct chemical sites for anchoring open active metals in subsequent studies, preventing open active metal sites from agglomeration and oxidation.

**Figure 5:**
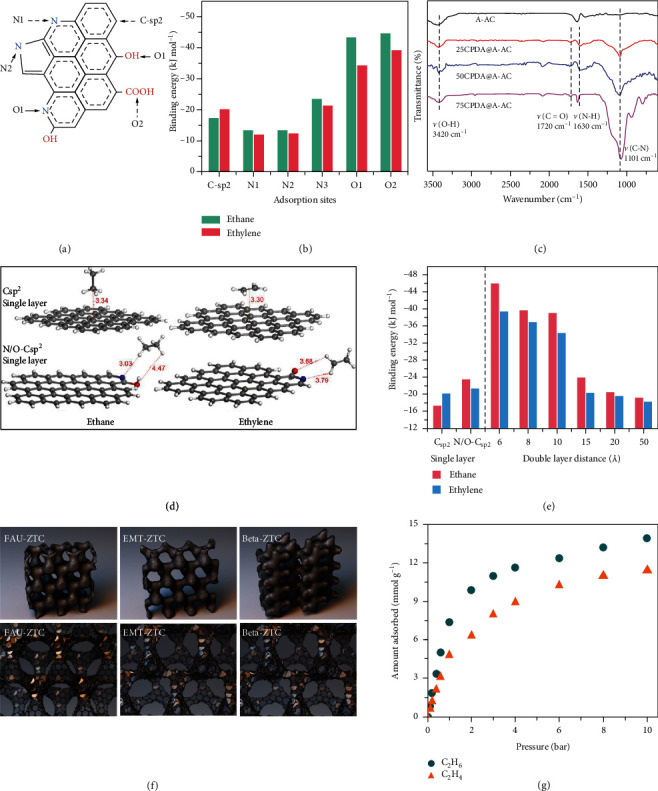
(a) Schematic illustration of O and N interaction sites in porous carbon and (b) corresponding binding energy of C_2_H_4_/C_2_H_6_ with different interaction sites. (c) FTIR spectra of samples [30]. (d) Interaction between C_2_H_4_/C_2_H_6_ and the C*sp^2^* and N/O-C*sp^2^* single layers. (e) The interaction energy between C_2_H_4_/C_2_H_6_ and different pore sizes of C*sp^2^* and N/O-C*sp^2^* double carbon layer is referred to as that of the C*sp^2^*, N/O-C*sp^2^* single carbon layers [98]. (f) Schematic structures and Schwarzite models of samples. (g) Adsorption isotherms of beta-ZTC at 303 K [96].

The C_2_H_6_-selective adsorption technology is considered a favorable strategy for energy saving in C_2_H_6_/C_2_H_4_ separation, which can directly afford high-purity C_2_H_4_. However, C_2_H_6_-selective adsorbents often have poor selectivity because of the insufficiency of adsorption sites. Although facing many challenges, the research on C_2_H_6_-philic carbonaceous adsorbents has been made great development in the past few years. For instance, to introduce adsorption sites with C_2_H_6_ affinity, Li and co-workers reported a novel composite porous carbon (CPDA@A-ACs) prepared by growing polydopamine (CPDA) on asphalt-based activated carbons (A-ACs) as a precursor [30]. With the increasing amount of CPDA loading, the O contents increased from 13.25 to 17.79%, and N contents increased from 3.07 to 4.06%. The bands of C=O, N-H and C-N can be observed in the CPDA@A-ACs composites rather than in the A-AC. This shows that the O/N concentrations increased by the CPDA loading. In addition, with the loading increases of CPDA, the pore size distributions of PDA@A-ACs are reduced and shift toward micropores. The 50PDA@A-ACs achieved the C_2_H_6_ uptake of 4.71 mmol g^−1^ and the C_2_H_4_ uptake of 3.81 mmol g^−1^ at 30 kPa and 298 K. The 50CPDA@A-ACs showed IAST selectivity of 3 for the C_2_H_6_/C_2_H_4_ binary mixtures (1/15, v/v). The Forcite module in Materials Studio calculations had confirmed that C_2_H_6_/C_2_H_4_ adsorption selectivity originated from the strong binding sites of C_2_H_6_, which are derived from C-H···*π*, CH···N/O (Figures 5(a)–5(c)). The binding energies followed the order of O sites > C-*sp^2^* sites > N sites, so the O functional groups were proved to play an important role in enhancing C_2_H_6_/C_2_H_4_ separation selectivity by comparing the binding energies. Similarly, the Li group reported another series of carbon >materials, which can selectively adsorb C_2_H_6_ efficiently [98, 99]. The underlying mechanism was interpreted by Molecular simulation. The result shows that C_2_H_6_ has *sp^3^* configuration, which could interact with O and N functional groups on the pore wall through H-bonding more efficiently (Figures 5(d) and 5(e)).

Previous reports showed that carbonaceous adsorbents can selectively adsorb C_2_H_6_ at low pressures (1 bar or less than 1 bar). From the perspective of engineering, it is important to enhance the C_2_H_6_ selective adsorption capacity of the adsorbent from the C_2_H_6_/C_2_H_4_ binary mixture at high pressures (10 bar or higher). Recently, Chang and co-workers introduced zeolites (FAU, EMT, and beta) as the templates for the synthesis of microporous three-dimensional (3D) graphene-like porous carbons with C_2_H_6_ selective [96]. The FAU-, EMT-, and beta-zeolite templated carbons (ZTC) have different pore sizes of 1.2, 1.1, and 0.9 nm, respectively (Figure 5(f)). Among them, the beta-ZTC is composed of numerous polygon graphene rings that provide different degrees of strain, which was indicated by ^13^C NMR, Raman, and XPS. Thus, the concentrations of other types of defects and a diverse range of polygons in beta-ZTC were proven to be higher than those in FAU- and EMT-ZTCs. Interestingly, the beta-ZTC with the most curved graphene structure showed preferential adsorption of C_2_H_6_ (14 mmol g^−1^) over C_2_H_4_ (11.2 mmol g^−1^) at 10 bar and 303 K (Figure 5(g)). Conversely, a graphene sheet without curvature does not achieve C_2_H_6_/C_2_H_4_ separation. Thus, the author thought that the negatively curved graphene structure could affect the C_2_H_6_/C_2_H_4_ separation performance. Not only that, unlike C_2_H_6_-selective carbon materials reviewed above, the C_2_H_6_-selective adsorption performance of beta-ZTC was not affected by controlling the oxygen content [30, 97, 98]. Breakthrough experiments showed that the directly obtained C_2_H_4_ purity is 99.9% at 5 bar.

### 4.3. C_3_H_6_/C_3_H_8_

Similar to the thermodynamic separation of other olefin/paraffin, C_3_H_6_ generally engages in stronger interactions with polar surfaces than C_3_H_8_ [100, 101]. Depending on the difference in pore microenvironment, porous carbons can effectively achieve the separation of C_3_H_6_ and C_3_H_8_. As mentioned before, the thermodynamic effect has been an effective factor in the selective separation of C_3_H_6_/C_3_H_8_. The majority of research has been dedicated to the preferential adsorption of C_3_H_6_ by *π*-*π* interactions between open metal active sites and olefin. For instance, Comroe and co-workers reported that Ag(I)-doped microporous carbons were synthesized by using furfuryl alcohol as a precursor [94]. The S_BET_ of the microporous carbons with Ag(I)-doping content of 0.7-2.5 at.% can achieve 915-1193 m^2^/g. The IAST selectivity of C_3_H_6_/C_3_H_8_ is in the range of 2.4~5. It suggested that the porous carbons with the Ag(I) content of 2.5 at.% more affinity towards C_3_H_6_. If Ag(I) was not enough, it will not capture enough C_3_H_6_ by *π*-*π* complexation. From the theoretical point of view, selective adsorption of C_3_H_6_ from C_3_H_6_/C_3_H_8_ mixtures was largely investigated in the framework of the Density Functional Theory (DFT). The DFT proved reliable results in reproducing binding energies and equilibrium geometries in good agreement with the experimental data since it allows for obtaining accurate information on the electron transfer during the adsorption process. For instance, Chakraborty and co-workers performed a combined experimental and theoretical study, explaining how Ag(I)-doped porous carbon can effectively separates C_3_H_6_/C_3_H_8_ briary mixture [31]. The frame of molecular orbital theory and DFT results indicated that the *d*-*π* complexation between the S-Ag(I) open active metal sites and olefin (C_2_H_4_ or C_3_H_6_) existed in pores. This interaction makes the adsorption energy between the olefins and paraffin a substantial difference. When the narrow pores contain paraffin, Ag(I) could interact with the *sp^2^-C* on the other side of the graphene plane, rather than paraffin. However, in presence of olefin molecules, *π*-*π* bond interaction occurs between Ag(I) and olefins, rather than *sp^2^-C* interaction in the graphene plane.

For the purification of feeds containing low concentration C_3_H_8_, porous carbon with an affinity for C_3_H_8_ will be an ideal adsorbent, which can directly obtain high-purity C_3_H_6_. For instance, Mendes and co-workers constructed novel carbonaceous adsorbents for C_3_H_8_/C_3_H_6_ separation, which had an excellent affinity for C_3_H_8_ over C_3_H_6_ [102]. The carbonaceous adsorbents were synthesized by using phosphoric acid-treated phenolic resin as a precursor. Next, the adsorbents were placed in the C_3_H_6_ atmosphere for 12 days. The phosphoric acid and C_3_H_6_ treatments led to changes in the surface chemistry properties of carbonaceous adsorbents. The adsorbents surface pretreated with phosphoric acid exists C=O and P-O-C functional groups, whereas C_3_H_6_ can be used as a cleaner of these O functional groups and produce C=C and P=O functional groups in porous carbon inner surface. Meanwhile, the C_3_H_6_ treatment can open the constriction pore to more straight interpore connections, promoting the kinetic diffusion of C_3_H_8_. Thus, the sample displays a higher uptake for C_3_H_8_ (2.9 mmol g^−1^) over C_3_H_6_ (1.4 mmol g^−1^) at 100 kPa and 25 °C.

### 4.4. Separations of C_4_ Isomer

To date, different types of adsorbents have been reported for the separation of C_4_ isomers, including MOFs, zeolites, and porous carbons [103–105]. Among them, the adsorption separation of C_4_ isomers is still in its infancy by using porous carbons, and a few publications had been reported to date. For instance, Xiao and co-workers synthesized a series of starch-based ultramicroporous carbon spheres by using the alkali metal ion-exchange method to control micropore size (SC-M; M = Na, K, Rb). The ultramicropore size of SC-M was tuned on the sub-Ångstrom level [70]. The potassium derivative SC-K can achieve high uptake of C_4_H_6_ at 100 kPa and 298 K (2.36 mmol g^−1^). The C_4_H_6_/n-C_4_H_8_ and C_4_H_6_/i-C_4_H_8_ adsorption capacity ratios of SC-K reached 3.75 and 4.72, respectively. The excellent selectivity of SC-K originates from extremely narrow micropores of 4.83 Å. In addition, the bicomponent breakthrough experiment results showed that the SC-K had an outstanding performance in the dynamic separation of C_4_ alkadiene/alkenes. On top of that, the adsorbent can be regenerated easily under He flow at 353 K.

Although research on the adsorption separation of C_4_ isomers by porous carbons is limited, there are some reports about carbon molecular sieving membranes (CMMSs) for butane isomer separation. For instance, Yang and co-workers introduced ultramicropores CMMSs, which were synthesized by pyrolysis of P84 polymer [106]. The slit-like ultramicropores of CMSMs were tailored on the Ångstrom level by fine-tuning the pyrolysis temperature. The best average separation factor of n-butane/iso-butane is 74 which emerged in CMMS obtained by pyrolyzing at 600 °C (CMMS-600). The CMMS-600 has ultramicropores centered at 6.0 Å which lies between the kinetic diameters of n-butane and iso-butane. Thus, it is a feasible design method for synthesizing the effective carbonaceous adsorbents by using the above CMMSs membranes precursors.

## 5. Separation Based on Kinetic Effect

Unlike equilibrium separation, the kinetic separation is reached by the differences in diffusion rates of gas mixtures. The size of the pore entrance plays a critical role in restricting the diffusion of certain adsorbates, leading to partial size exclusion [107, 108]. In this section, we highlighted the main research progress of LHs kinetic separation using porous carbon. We summarized and compared several best-performing porous carbons in kinetic separation.

### 5.1. CH_4_/N_2_

Taking advantage of the difference in molecule kinetic diameters of N_2_ (0.36 nm) and CH_4_ (0.38 nm) molecules can achieve separation. The purification can be effectively achieved through precise control of the pore size/shape to make CH_4_ diffuse in porous carbons, but it significantly restricts the diffusion of the N_2_ molecules [109–112]. For instance, Zhang and co­workers prepared modified CMS materials with a chemical vapor deposition method to decrease the micropore size of activated carbon [113]. By precisely controlling the deposition temperature, time, and flow rate of benzene, the CMS pore size decreased to form an aperture ideally sized to accommodate N_2_ but not CH_4_. As a result, the CMS-G showed a high CH_4_/N_2_ kinetic selectivity (35.26) and a good performance in the PSA experiment (enrichment of 30.20% for CH_4_). Besides, for the CMS, it is necessary to consider the barrier resistance at the entrance of the micropore and the pore diffusion resistance for N_2_/CH_4_ separation, as proposed by Yang and co-workers [114]. The authors used the dual resistance model to determine the adsorption kinetics of binary gas mixtures by batch uptake experiments. It indicated that the adsorption rate of binary gas mixtures can be controlled by the surface barrier resistance at the entrance of the micropore and diffusion in the micropore interior. The transport of CH_4_ (6.04∗10^7^ s^−1^) is much slower than that of N_2_ (1.21∗10^10^ s^−1^). Accordingly, although CH_4_ breaks through together with N_2_ in a breakthrough experiment, the CH_4_ was hardly adsorbed because of the slower diffusion transport than N_2_.

### 5.2. CH_4_/CO_2_

As for CH_4_/CO_2_ separation, CO_2_ is easier to diffuse into the pores because of its smaller kinetic size (0.33 nm) than that of CH_4_. The adsorption performances of CO_2_ and CH_4_ on a commercial CMS were reported by Rocha and co-workers [115]. The CMS can effectively trap CO_2_ and impede the diffusion of CH_4_, to achieve kinetic and thermodynamic combined selectivity. The diffusion of gases in the adsorbent clearly showed that CO_2_ diffused through the micropores much faster than CH_4_. It indicated that the constriction at the entrance of the micropore has an important contribution to the overall diffusion resistance. The equilibrium for CO_2_ was reached after approximately 800 s, while the equilibrium for CH_4_ was reached over 25 h. The CO_2_ and CH_4_ diffusion constant were calculated to be 0.01 and 0.0001 s^−1^, respectively. Kapoor and co-workers evaluated the CH_4_/CO_2_ kinetic separation performance by using a carbon molecular sieve [116]. It is shown that the CO_2_ with stronger adsorption has a quicker diffusion rate and the diffusion time constant is 9.7∗10^−4^s^−1^. On the contrary, the CH_4_ has a slower diffusion rate, and the diffusion time constants were calculated to be 5∗10^−6^s^−1^. The CO_2_/CH_4_ diffusivity rate ratio was 180 at 298 K. This is beneficial for CH_4_/CO_2_ kinetic separation, particularly when CH_4_ was the desired product.

### 5.3. C_3_H_6_/C_3_H_8_

**Figure 6:**
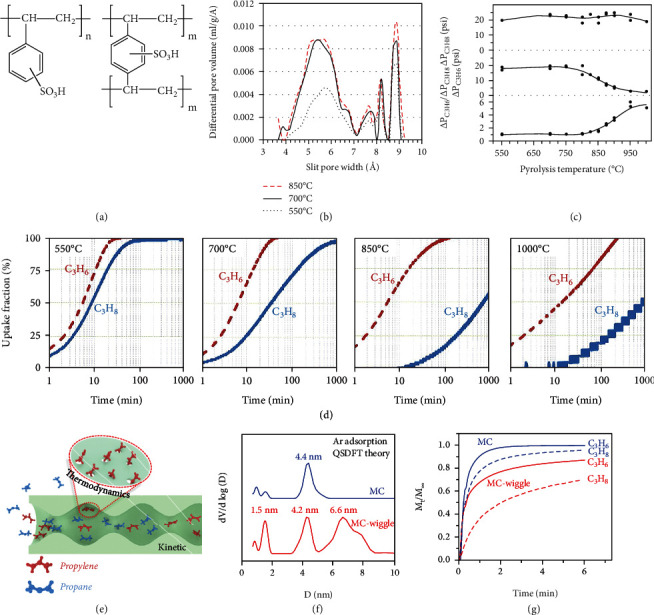
(a) Simplified chemical structure of cation exchange resin: monosulfonated polystyrene. (b) Pore size distribution. (c) The pressure drops of C_3_H_6_ (top) and C_3_H_8_ (middle) and the ratio of the two (bottom) during adsorption at different carbonization temperatures. (d) Time-resolved adsorption curve for CMS samples with different carbonization temperatures [117]. (e) Illustration of the proposed mechanism for C_3_H_6_/C_3_H_8_ separation in MC-wiggle. (f) Pore size distributions. (g) Time-resolved absorption curves for C_3_H_6_/C_3_H_8_ at 298 K [119].

Analogous to other LHs separation, the pore aperture size/shape of carbon materials play a key role in achieving the kinetic separation of C_3_H_6_/C_3_H_8_. For instance, Liu and co-workers prepared a separation of C_3_H_6_/C_3_H_8_ novel CMS, which was synthesized through pyrolysis a gel-type strong acid cation exchange resin [117] (Figures 6(a) and 6(b)). With the pyrolysis temperature enhanced from 700 to 850 °C, the adsorption half-time ratio (t_0.5_(C_3_H_8_)/t_0.5_(C_3_H_6_)) increased from 1 to more than 100, and the diffusion rate of the C_3_H_8_ was noticeably slower. The C_3_H_6_/C_3_H_8_ kinetic selectivity (90) is similar to that of zeolite 4A, while the C_3_H_6_ diffusion rate is 30 times faster than that of zeolite 4A. However, the diffusion ratio of C_3_H_6_ is slowed down for carbon materials pyrolysis at 1000 °C, which is not conducive to the kinetic separation of C_3_H_6_/C_3_H_8_. By comparing the pressure change (*Δ*P) from start to finish of adsorption in a closed environment, the authors further explored the effect of pyrolysis temperatures on the adsorption capacity of C_3_H_6_ and C_3_H_8_. Since the pore size of carbon materials gets narrowed as pyrolysis temperature increases, the pore volume close to the size of C_3_H_8_ with a larger molecular diameter is reduced, but close to that of the C_3_H_6_ changes little (Figures 6(c) and 6(d)). Recently, Liu and co­workers further systematically studied the separation performance of C_3_H_6_/C_3_H_8_ on the polyvinylidene chloride copolymer (PVDC) derived porous carbons [118]. This work can precisely control the micropore size of PVDC-derived CMS by choosing polymer precursor and pyrolysis temperature. With the pyrolysis temperature increased from 700 to 1500 °C, the effective micropore size can decrease to 6.2-4.2 Å. The CMS-18 (1500°C pyrolysis) showed an obvious difference in the diffusivity rate of C_3_H_6_/C_3_H_8_, and the diffusivity of C_3_H_6_ (D/r^2^ =1.6∗10^−5^s^−1^) was 123 times faster than C_3_H_8_ (D/r^2^ =1.3∗10^−7^s^−1^), because the pore aperture is 4.2 Å, which can block C_3_H_8_ adsorption. The C_3_H_8_ did not reach equilibrium even after 20 days.

Our group recently demonstrated a new strategy to create wiggling mesopores in monolithic carbon (MC-wiggle) through a perturbed self-assembly method [119]. By such nanoscale mesopore geometry engineering, we achieved a C_3_H_6_/C_3_H_8_ separation selectivity of around 39.0 and a C_3_H_6_ uptake of 2.6 mmol g^−1^, which outperform not only the reported carbonaceous adsorbents but also the top-performing crystalline adsorbents like MOFs and zeolites. Fundamentally, this is the first wiggling transport behavior observed on mesoporous carbon materials, which featured a unique two-step desorption process for all the gas probes in terms of Ar, N_2_, H_2_O, and toluene. Besides the gas adsorption techniques, the state-of-the-art electron tomography was applied to reconstruct the 3D wiggling mesoporous channels. Based on this, we proposed that the bigger C_3_H_8_ molecules were easily colliding with and bouncing back the geometrical bulges in the pore channel, resulting in a significantly lower diffusion rate of C_3_H_8_ (2.1∗10^−4^s^−1^) in the MC-wiggle, while the smaller C_3_H_6_ (4.2∗10^−3^s^−1^) were less affected, thus amplifying the kinetic selectivity. It is different from the common thermodynamic effect that relies on strong binding sites. This is the first wiggling transport-induced kinetic enhanced C_3_H_6_/C_3_H_8_ separation (Figures 6(e)–6(g)).

## 6. Structured Carbon Adsorbents

Carbon adsorbents are often produced as powders that need post-shaping by pressurizing or using binders before their practical applications [120, 121]. In comparison to powders, pellet adsorbents exhibit greater operational flexibility. Microscopically, the 3D continuous hierarchical structures warrant multiple advantages such as fast heat and mass transfer, low pressure drop, and high contacting efficiency [122, 123]. The shaping methods in terms of extrusion, colloidal processing, coatings of honeycombs, etc. are widely explored, where normally require organic or inorganic binders. Binder-free synthesis represents a research frontier. For instance, our group developed a series of carbon pellets derived from functional polymer precursors by a one-step sol-gel method [59, 124, 125]. Although these carbon adsorbents showed a hierarchically porous structure, the connectivity of the mass transport channels can be further improved.

In addition, the development of complex-shaped carbon adsorbents is another research direction. For instance, 3D printing technique enables more options for the geometric design of adsorbent pellets, which can be directly used in adsorption processes. Thus, this technique has been employed as a viable option for the production of shaped carbon adsorbents [126]. Recently, Garnica and co-workers reported that sponge-like carbons with tailored channel architecture and porosity were prepared by combined sol-gel polymerization and 3D printing technology [127]. Mendes and co-workers prepared composite adsorbents composed of porous carbon and 13X zeolite, which were then 3D printed using bentonite as a binder [128]. However, the use of 3D printed porous carbon adsorbents for LHs separation is rarely reported, which could be a developing trend in future.

## 7. Conclusions and Perspectives

Porous carbon materials have attracted tremendous research interest for LHs separation in recent years, and the increasing number of publications on this topic signifies its importance. As compared with other types of adsorbent materials, carbon-based materials could play a bigger role in LHs separation, particularly in the aspect of practical applications. It shows a great variety in structural modulation which includes its pore structure (size, geometry), morphology (sphere, sheet, fiber), and chemical properties. In addition, doping heteroatoms into carbon matrix can also well modify their separation factor due to the enhanced interaction between the decorated adsorption sites and the specific gas. The full combination of open active metal sites (such as Cu^+^ and Ag^+^) and porous carbon provides a good choice to further promote the carbonaceous adsorbent of olefin selectivity. Furthermore, scientists should pay more attention to the gas diffusion behaviors in slit-type micropores and consider the separation performance from a kinetic point of view. Despite the available progress, there is still a huge demand for the development of advanced carbonaceous adsorbents for LHs separation in the chemicals industry. We listed the main issues and/or developing trends in this field as follows:

1. Judiciously integrating porous carbons with other functional materials (e.g., zeolites, MOFs, COF, ZIF, and POPs) would mitigate the drawbacks of the individual components and provide synergistic effects to enhance LHs separation performance. The unique carbon composites can better achieve separate those multiple and complex components mixtures, such as C1-C4 mixed gas. It has been reported that loading functional materials in porous carbon or physically mixing can quickly construct functional carbonaceous adsorbents. However, if the functional materials are introduced during the precursor synthesis stage, more precise control and design will be achieved, which is more challenging. Because the high-temperature carbonization process will destroy the structure of functional materials, thus the use of functional material with high thermal stability may be a good choice. In conclusion, scientists need to propose more novel and efficient strategies to construct a composite structure to improve the comprehensive performance of carbonaceous adsorbents
2. To date, the control of pore shapes of carbonaceous adsorbents has not been paid enough attention. It is precisely the subtleties in the local geometries that have a profound influence on the gas behavior properties. However, porous carbon with unusual silt microporous morphology is built from disorganized graphene-like planes, which is difficult to achieve the same flexible design as crystalline materials at the molecular scale. Thus, the key to the slit pore size regulation lies in the stacking parameters between the graphite crystallites, which include the angle, the degree of bending, and the continuity. The current strategy is still difficult to precise control for units of single-layer graphite, including chemical activation, chemical deposition, and control of the carbonization process. Carbon scientists should focus more on the gradient control of the molecular structure in the chemical synthesis of carbon precursors, such as the number of benzene rings, carbon-chain lengths, and group positions. It may be a very potent strategy to precisely control the stacking parameters between graphite layers through differences in molecular structure, which can achieve narrow ultramicropores in carbon materials with sub-Ångstrom precision
3. Researchers have made efforts trying to quantify the role of heteroatom doping for carbon adsorbents. The selective adsorption of LHs molecules over carbons with less functional groups mainly depends on matched pore structure, such as pore size, geometry, and connectivity. In this case, it is a surface coverage and micropore filling process. The gas adsorption isotherm usually has a lower adsorption capacity at low pressure than carbon materials with preferential adsorption sites, due to the lack of strong interaction. However, for the thermodynamic mechanism, further research is needed to unveil the contribution of nanopores and surface functional groups to the selective adsorption of targeted hydrocarbons
4. Olefin and CH_4_ are the desired product; the reversed paraffin/impurity gas selective (“reverse” selective) materials would be more favorable. The “reverse” selective carbonaceous adsorbents will greatly simplify the separation process and reduce the energy consumption for adsorbent regeneration as well as enhance gas purity. Thus, the further development of the “reverse” selectivity of carbonaceous adsorbents is also an area that requires deeper research and insight into the separations of LHs. For instance, the generality of the “reverse” selective of C_2_H_6_/C_2_H_4_ needs to be further clarified. Why do carbonaceous adsorbents without open metal sites generally have higher adsorption capacity for C_2_H_6_ than for C_2_H_4_? Can the separation of C_2_H_4_ and C_2_H_6_ be achieved by kinetics or size sieving effect?
5. Comprehensive and systematic characterizations will be of great help for observing gas adsorption behaviors and the evaluation of adsorbents practical performance. The adsorption of LHs/impurity gases in micropores can be obtained by gravimetric or volumetric methods, thereby revealing the overall state of the adsorbed LHs/impurity gases. However, the status of LHs/impurity gas in confined slit pores and their interaction with specific adsorption sites on the pore walls is still elusive. Thus, it highly requires advanced characterizations, particularly in situ techniques (e.g., in situ NMR and inelastic neutron scattering) with molecular level even atomic resolution to probe LHs/impurity gas adsorption behaviors in the subnanometer pores
6. For industrial applications, many factors must be considered, such as stability, scale-up, cost, and multicycle renewability. Under stringent criteria of industry, we have to point out that the reported most adsorbents are in the basic level to demonstrate the laboratory-scale viability, without considering practical applications. Therefore, future exploration must address the “performance parameters spectrum” relevant to commercial applications, rather than blindly pursuing LHs separation performance

All in all, porous carbons can exhibit unparalleled LHs separation and purification performances because of their intrinsic structural advantages and hold great promise for practical applications. Of course, these goals require interdisciplinary know-how input, by profiting knowledge from other disciplines, including organic chemistry, material science, and solid-state chemistry. We have a reason to believe that foreseeable improvements will be made in the LHs separation community in near future.

## Figures and Tables

**Table 1:** Physical properties of light hydrocarbons of C_1_ to C_4_ [4].

Adsorbate	Boiling point (K)	Kinetic diameter (Å)	Dipole moment (×10^18^esu cm)	Polarizability (×10^−25^cm^3^)	Application background
CH_4_	111.6	3.8	0	25.9	Natural gas purification
C_2_H_6_	184.6	4.4	0	44.3	Producing high-purity ethylene
C_2_H_4_	169.4	4.2	0	42.5	
C_3_H_8_	231.0	4.3-5.1	0.084	62.9	Producing high-purity propylene
C_3_H_6_	225.5	4.7	0.366	62.9	
n-C_4_H_10_	272.7	4.7	0.05	82.0	Paraffin separation of normal paraffin from iso-paraffins
i-C_4_H_10_	261.3	5.3	0.132	81.4-82.9	
n-C_4_H_8_	266.9	4.5	0.359-0.438	79.7-85.2	Olefin separation
i-C_4_H_8_	266.3	4.8	0.501	78.9	
C_4_H_6_	268.6	5.2	0	86.4	Removal of dienes from olefins

**Table 2:** Summary of selected carbonaceous adsorbents for CH_4_ purification.

Sample ID	Uptake (mmol g^−1^)			Q_st_ (kJ mmol^−1^)		Selectivity		Ref.
	CH_4_	N_2_	CO_2_	CH_4_	CO_2_	S_CH4/N2_	S_CO2/CH4_	
PCNPs^a^	1.17	0.28	—	22.3	—	10^d^	—	[77]
GOC-2^a^	1.82	0.5	—	22.1	—	5.8^e^	—	[78]
SC-6^a^	1.86	0.6	—	24.9	—	5.7^e^	—	[79]
PRC-850^a^	1.12	0.38	—	23	—	5.4^d^	—	[80]
PS^b^	1.38	0.45	3.63	—	—	3.1	15^g^	[81]
GL^b^	0.45	0.17	1.65	—	—	2.7	54^g^	
OTS-1-550^a^	1.12	0.3	2.8	25	37.3	5.9^e^	7.4^g^	[82]
OTS-2-650^a^	1.67	0.5	3.96	24.7	33.8	5.2^e^	4.1^g^	
OTSS-3-350^a^	0.9	0.26	2.83	—	39.2	5.6^e^	9.5^i^	[83]
OTSS-2-450^a^	0.93	0.3	2.94	—	47	4.9^e^	12.8^i^	
CGUC-0.5-6^a^	0.9	0.2	3.4	—	31	6.7^e^	34.9^h^	[84]
CGUC-1-6^a^	1.01	0.2	3.26	—	27.5	5.9^e^	16.9^h^	
SNMC-1-600^a^	1.45	0.5	3.98	—	27.5	5.1^e^	6.9^h^	[74]
SNMC-2-600^a^	1.57	0.5	4.24	—	26.4	4.2^e^	4.3^h^	
SNMC-3-600^a^	1.17	0.4	3.0	—	23.3	3.6^e^	3.2^h^	
Co-NDPC-500^a^	1.0	0.26	3.5	27	35	3.9^f^	10.4^e^	[85]
Co-NDPC-600^a^	1.2	0.27	5.3	28	53.8	4.4^f^	11.4^e^	
CC-PNP^c^	2.20	—	3.7	—	—	—	15.9^i^	[86]
P-CC-PNP^c^	4.26	—	7.5	—	—	—	5.2^i^	
C30-CC-PNP^c^	7.02	—	11.9	—	—	—	4.3^i^	
C85-CC-PNP^c^	5.35	—	8.9	—	—	—	4.8^i^	
Chit_supCO_2__P800^a^	0.01	—	1.37	—	—	—	137^j^	[87]
Chit-PMO_P800^a^	0.01	—	0.30	—	—	—	30^j^	
Chit-PMO_P800_crushed^a^	0.14	—	1.01	—	—	—	7.2^j^	
C1000^a^	1.75	—	3.7	17.4	20.7	—	28^e^	[88]
PHA^a^	0.63	—	1.6	22.5	33.5	—	7.2^e^	[89]
NAHA-1^a^	1.65	—	3.75	23	29.7	—	7^e^	
NAHA-2^a^	1.5	—	4	23.5	29	—	6^e^	

Gas uptake at ^a^298 K and 100 kPa, ^b^313 K and 100 kPa, and ^c^298 K and 20 bar. IAST predicted selectivity for separation of CH_4_/N_2_ with mole ratios of ^d^30/70 and ^e^50/50. ^f^Uptake ratio of CH_4_/N_2_ as separation selectivity. IAST predicted selectivity for separation of CO_2_/CH_4_ with mole ratios of ^g^50/50, ^h^40/60, and ^i^10/90. ^j^Uptake ratio of CO_2_/CH_4_ as separation selectivity.

**Table 3:** Summary of the selected carbonaceous adsorbents for C_2_H_4_/C_2_H_6_ separation.

Sample ID	Uptake (mmol g^−1^)		Q_st_ (kJ mmol^−1^)		Selectivity S_C2H4/C2H6_	Ref.
	C_2_H_4_	C_2_H_6_	C_2_H_4_	C_2_H_6_		
CMK-3^a^	2.9	3.1	20	19	0.94^c^	[32]
4CuCl/CMK-3^a^	3.2	1.7	53	15	1.88^c^	
8CuCl/CMK-3^a^	3.6	1.3	66	13	2.77^c^	
MC-S-Ag-3^b^	3.4	2.6	—	—	2.4^d^	[94]
CuCl(6.0)/AC^a^	2.7	0.8	—	—	52^d^	[95]
CuCl(8.0)/AC^a^	2.6	0.7	—	—	69.4^d^	
beta-ZTC-O 2.2^a^	4.9	7.3	17	24	1.5^e^	[96]
EMT-ZTC^a^	4.1	5.9	24	25	1.6^e^	
FAU-ZTC^a^	4.0	5.0	28	25	1.7^e^	
25CPDA@A-ACs^b^	5.9	7.2	—	—	2.75^f^	[30]
50CPDA@A-ACs^b^	6.3	7.1	32	35	3^f^	
75CPDA@A-ACs^b^	5.9	7.1	—	—	2.79^f^	
NAC-800^b^	4.5	5.8	22	24	0.77^e^	[90]
ANPC-1-800^b^	5.7	6.8	30	34	1.54^e^	[97]
MGA-700-4^b^	6.0	7.3	24	29	6.76^f^	[98]
MGA-750-3^b^	5.7	7.0	22	28	6.44^f^	
C-PDA-3^b^	5.1	6.6	22	22	1.83^e^	[99]

Gas uptake at ^a^303 K and 100 kPa and ^b^298 K and 100 kPa. ^c^Uptake ratio of C_2_H_4_/C_2_H_6_ as separation selectivity; IAST predicted selectivity for separation of C_2_H_4_/C_2_H_6_ with mole ratios of ^d^50/50; IAST predicted reverse selectivity for separation of C_2_H_4_/C_2_H_6_ with mole ratios of ^e^50/50 and ^f^15/1.
